# Professor Charles A. Lee, M. D., in Nashville, Tennessee

**Published:** 1868-08

**Authors:** 


### Professor Charles A. Lee, M. D., in Nashville, Tennessee.

At a meeting of the University Convocation of the State of New York, held on the 4th, 5th and 6th days of August, 1868, Professor Charles A. Lee, M. D., of the University of Buffalo, Medical Department, was appointed (with others) to represent the Convocation at the American Teachers' Association, to be held at Nashville, Tennessee, on the 19th, 20th and 21st days of August, 1868.

We notice in the report of the Convention, published in the *Nashville Republican Banner*, that Professor Lee is doing good service in the Convention, and that he "delivered an address on school hygiene, in which he severely criticised the pernicious practice of students in schools and colleges spending their spare time in idle lassitude, and indulging in the deleterious habits of chewing and smoking tobacco, and in other no less wicked amusements."

The address was listened to, not only by the Convention of Teachers, but by many of the principal citizens of the city; it was received with much favor and elicited a lengthy and interesting discussion. We suggest that Prof. Lee deliver the same address nearer home; it might answer schools and citizens of New York as well.

